# Serum and Cerebrospinal Fluid Malondialdehyde Levels in Patients with Mild Cognitive Impairment

**DOI:** 10.3390/jox15020050

**Published:** 2025-03-30

**Authors:** Stavroula Ioannidou, Argyrios Ginoudis, Kali Makedou, Magda Tsolaki, Evgenia Lymperaki

**Affiliations:** 1 Department of Biomedical Sciences, International Hellenic University, 57400 Thessaloniki, Greece; stayroyla.ioannidou@gmail.com; 2 School of Veterinary Medicine, Aristotle University of Thessaloniki, 54627 Thessaloniki, Greece; 3 Laboratory of Biochemistry, AHEPA University Hospital, School of Medicine, Aristotle University of Thessaloniki, 54636 Thessaloniki, Greece; kalimakedou@gmail.com; 4 Greek Association of Alzheimer’s Disease and Related Disorders (GAADRD), 54248 Thessaloniki, Greece; tsolakim1@gmail.com; 5 Laboratory of Neurodegenerative Diseases, Center for Interdisciplinary Research and Innovation (CIRI), Aristotle University of Thessaloniki, 54124 Thessaloniki, Greece

**Keywords:** lipid peroxidation, malondialdehyde, oxidative stress, mild impairment cognitive, cerebrospinal fluid biomarkers

## Abstract

Mild cognitive impairment (MCI) is recognized as an intermediate stage between normal aging and dementia. Oxidative stress is implicated in the pathophysiology of neurodegenerative diseases, playing a crucial role. This study aimed to investigate the differences in malondialdehyde (MDA) levels in the serum and cerebrospinal fluid (CSF) of patients with MCI compared to FDA-approved biomarkers, based on age, sex, and education level. Participants aged 55–90 years old were categorized into three groups based on FDA-approved biomarkers, especially the CSF Aβ42/Aβ40 ratio and clinical screening assessments: 30 MCI (A+) patients with abnormal CSF Aβ42/Aβ40 ratios (Group A), 30 MCI (A−) patients with normal CSF Aβ42/Aβ40 ratios (Group B), and 30 healthy (A−) participants with normal CSF Aβ42/Aβ40 ratios (Group C). The measurements of CSF FDA-approved biomarkers were performed using an automated immunochemical method (Fujirebio, Inc.), while MDA determination was performed using a competitive inhibition enzyme immunoassay technique (ELK Biotechnology Co., Ltd.). Our results showed that the mean CSF MDA values were significantly lower in group C than in group A (83 ng/mL vs. 130 ng/mL, *p* = 0.024) and group B (83 ng/mL vs. 142 ng/mL, *p* = 0.011), respectively. Differences in serum and CSF MDA levels were presented in the study groups based on sex, age, and education level. These findings suggest that lipid peroxidation, as indicated by CSF MDA, could serve as a potential biomarker for the early recognition of MCI.

## 1. Introduction

Alzheimer’s disease (AD) is the most common neurodegenerative disorder and the leading cause of dementia in the elderly. It is estimated that 153 million people globally will be living with AD by 2050 [1]. In the coming years, AD will become a major public health issue in countries with extended life expectancy. The number of people living with AD or another form of dementia is expected to rise significantly due to the increased risk associated with advanced age [2].

Mild cognitive impairment (MCI) is a condition characterized by cognitive decline greater than expected for a person’s age and education level, without affecting their ability to perform routine daily tasks [3]. A significant percentage of individuals with MCI may remain stable or regain normal neurological function, while only 10% to 15% may develop dementia within a year [4,5]. MCI is distinguished into two subtypes, based on screening assessments, amnestic (aMCI), commonly associated with high risk of developing AD, and non-amnestic (nMCI), which is less likely to lead to AD [6]. Additionally, based on CSF biomarkers, MCI can be distinguished as MCI (A+), characterized by an abnormal CSF Aβ42/Aβ40 ratio indicative of underlying AD, or MCI (A−), which has a normal CSF Aβ42/Aβ40 ratio, suggesting a lower likelihood of AD progression [7,8]. The degeneration of neurons in specific regions of the hippocampus and entorhinal cortex mainly occurs in MCI and the early stages of AD. The degeneration of neurons is caused by the extracellular deposition of amyloid β (Aβ), known as senile plaques, and intracellular hyperphosphorylation of tau protein in neurons [9]. MCI is often considered an intermediate stage between normal aging and AD or a prodromal phase of AD [5,10].

According to the National Institute on Aging and the Alzheimer’s Association, the developing criteria for the symptomatic predementia phase of AD or MCI include clinical examination, neuroimaging methods, and cerebrospinal fluid (CSF) biomarker analysis [11]. A classification system is proposed, referred to as the A/T/N system. Class “A” corresponds to the Aβ biomarker, class “T” refers to the phosphorylated tau protein (p-tau) biomarker, and class “N” stands for neurodegeneration. Each class can be rated as positive or negative. Particularly, a positive Aβ biomarker, a positive p-tau marker, and/or a positive neurodegeneration biomarker are required to identify the MCI individuals at high risk of progressing to AD [9,12]. 

Based on the Alzheimer’s Association, around 12% to 18% of people over the age of 60 are living with MCI [12,13]. Especially, among elderly women, the prevalence of MCI is higher compared to men, and the longitudinal rates of cognitive decline are more pronounced in women with MCI relative to men with MCI [14,15]. A study’s sex analysis found a protective effect against MCI in males, whereas it found no significant association with dementia risk [16]. Education is considered to be a significant predictor and protective factor against cognitive impairment, while higher education is related to greater cognitive activity and lower risk, regardless of sex or professional complexity [16,17,18]. Serum or CSF MDA levels may provide critical insights into MCI and highlight potential biomarkers that represent oxidative damage and cognitive dysfunction.

The absence of effective treatments capable of reversing or preventing the biological changes in the brain of patients exacerbates the burden of MCI and AD. The socioeconomic impact on patients and their families is about to reach serious dimensions [19,20]. Early diagnosis of MCI is, therefore, essential to delay mental deterioration and mitigate the socioeconomic impact of AD. Studies aim to identify more accurate, cost-effective, and less invasive blood-based diagnostic biomarkers to detect patients with MCI at high risk of developing AD or another form of dementia [21,22,23,24].

Research studies support the involvement of oxidative stress as a primary and critical factor in the pathophysiology of MCI and AD. Oxidative stress, mainly caused by the accumulation of Aβ oligomers in the inner mitochondrial membrane, leads to disruption of the electron transport chain and a reduced ability of antioxidants to limit oxygen free radicals. This results in DNA damage, protein oxidation, and lipid peroxidation. Oxidative damage indices, among others, include markers of lipid peroxidation. Lipid peroxidation comprises the oxidative degradation of lipids in cellular membranes, leading to the generation of reactive aldehyde species, such as malondialdehyde (MDA) [25]. Elevated levels of MDA have been implicated in impairing the integrity and function of neurons, contributing to cognitive decline in MCI [26].

The aim of this study is to evaluate and compare the levels of MDA with FDA-approved biomarkers, according to age, sex, and education, in two different biological fluids, serum and cerebrospinal fluid. In this study, participating patients with mild cognitive impairment were divided into two groups: MCI (A+) patients who are amyloid-positive and MCI (A−) patients who are amyloid-negative, along with healthy controls. This study aims to investigate the potential use of MDA as an additional biomarker in serum and CSF in order to distinguish MCI patients.

## 2. Materials and Methods

### 2.1. Study Population

In this study, a sample of 90 adults was included, 48 females and 42 males, aged 55–90 years old. All participants were research volunteers for the Greek Association of Alzheimer’s Disease and Related Disorders (GAADRD). The database of GAADRD includes participants diagnosed with conditions like normal cognition, mild cognitive impairment (MCI), Alzheimer’s disease (AD), and other related disorders, based on standardized clinical assessments.

The inclusion criteria were established for all participants, including healthy controls, according to screening assessments, medical history, laboratory tests, and physical examinations. The healthy controls participated as research volunteers. Some of them had a family history of dementia, although they did not exhibit any symptoms. The participants were examined by a neurologist, who followed the screening assessments, including (a) Mini-Mental State Examination, an assessment test for global cognition [27], (b) the functional rate scale for symptoms for dementia, a test for functionality [28], and (c) the Hamilton assessment test for depression [29]. Also, all personal information, such as age, sex, and education, along with physical and neurological examination findings, was recorded by the committed neurologist of GAADRD.

Initially, participants were divided into 60 patients with MCI and 30 healthy controls, based on screening assessments. Subsequently, they were further categorized according to CSF amyloid into three groups: 30 MCI (A+) patients with an abnormal CSF Aβ42/Aβ40 ratio (Group A), 30 MCI (A−) patients with cognitive decline and a normal CSF Aβ42/Aβ40 ratio (Group B), and 30 healthy (A−) controls with a normal CSF Aβ42/Aβ40 ratio (Group C). More specifically, the cut-off points for determining positivity were an Aβ42/Aβ40 ratio <0.058, accompanied by at least two other biomarkers: Aβ42 < 638 pg/mL, Aβ40 > 11,017 pg/mL, t-tau > 404 pg/mL, and p-tau > 52.1 pg/mL [30]. Individuals with positive CSF biomarkers are classified as MCI (A+) and are at an increased risk of developing AD in later years. All participants were not currently on any medication, supplement intake, or other drugs.

The characteristics of the study groups are presented in terms of sex, age, body mass index (BMI), education level, and family history in the following table (Table 1). Sex was categorized as male and female within the study groups. Age was classified as either under or over 74 years old to compare middle-aged individuals (55–74 years old) with older individuals (over 74 years old). Alzheimer’s disease is considered an age-related condition, with individuals ≥74 years being more likely to develop dementia, whereas individuals <74 years old might be in the early stages of cognitive decline [2,31]. Education level was categorized into primary, secondary, and tertiary.

### 2.2. Sample Collection

All serum and CSF samples from the participants were collected from the sample bank of the Greek Association of Alzheimer’s Disease and Related Disorders, following approval of this study by the bioethics committee. The samples were collected, isolated, and stored at −80 °C. Hemolyzed samples were excluded. Prior to the experiment, all samples, serum and CSF, were incubated at room temperature. Sterile solutions and disposable consumables were used in all determinations, and standard laboratory practices were followed for the safe handling of samples, laboratory space, and personal protection.

### 2.3. Cerebrospinal Fluid FDA-Approved Biomarkers Analyses

The analysis of Aβ40, Aβ42, p-tau, and t-tau protein levels in CSF was conducted using an automated immunochemical method, using the Lumipulse G1200 automated analyzer (Fujirebio Inc., Tokyo, Japan). The FDA-approved biomarkers were assessed according to the biobank’s protocol prior to patient selection.

### 2.4. Malondialdehyde Analysis

The MDA assay utilizes a competitive inhibition enzyme immunoassay technique, using ELISA test kits from ELK Biotechnology Co., Ltd. (Denver, CO, USA). A commercially available 96-well microtiter plate pre-coated with Human MDA protein was used. Standards and samples were added to the appropriate microtiter plate wells, followed by the addition of a biotin-conjugated antibody specific to Human MDA. Then, the plate was incubated at 37 °C for one hour and washed three times. Avidin conjugated to horse radish peroxidase was added, followed by another one-hour incubation and five consecutive washes. Subsequently, the TMB substrate solution was added. The enzyme–substrate reaction was terminated by the addition of sulfuric acid solution, and the color change was measured spectrophotometrically at a wavelength of 450 nm. The MDA concentration in the sample was calculated based on a standard curve.

### 2.5. Statistical Analyses

For the statistical analyses, we used a statistical software package (IBM Corp., released 2021; IBM Statistical Package for Social Sciences—SPSS for Windows, Version 28.0, Armonk, NY, USA). Descriptive statistics were performed for each sociodemographic variable. The sample data were checked for normality with the Shapiro–Wilk test, and appropriate statistical tests were chosen accordingly for analyses. The Student *t*-test for independent samples was used to investigate the differences in the mean values of the measured variables between the three study groups. For all statistical analyses, the level of significance was set at *p* < 0.05.

### 2.6. Ethical Considerations

The study was conducted in accordance with the Good Clinical Practice guidelines and the Declaration of Helsinki. Ethical approval of the study was obtained from the bioethics committee of the Greek Society for Alzheimer’s Disease and Related Disorders 175/2024 AI, 4 July 2024. All participants provided informed consent for the use of their samples in research. The confidentiality of the participants was strictly preserved, and personal privacy was fully respected.

## 3. Results

Table 2 presents the mean values of CSF FDA-approved biomarkers, CSF MDA, and serum MDA in three groups: Group A: MCI (A+), Group B: MCI (A−), and Group C: control (A−). The statistical analysis revealed significant differences in mean values of Aβ42, Aβ42/Aβ40 ratio, and t-tau among the study groups. Aβ42 levels were highest in healthy controls (1237 pg/mL), followed by MCI (A−) patients (1004 pg/mL), whereas MCI (A+) patients had markedly lower levels (590 pg/mL). The Aβ42/Aβ40 ratio was highest in healthy controls (0.10), followed by MCI (A−) patients (0.09), whereas MCI (A+) patients had markedly lower levels (0.05). T-tau levels were highest in MCI (A+) patients (633 pg/mL), followed by MCI (A−) patients (314 pg/mL), whereas healthy controls had markedly lower levels (252 pg/mL). The mean values of Aβ40 showed a significant difference between MCI (A+) and MCI (A−) patients (13,087 pg/mL vs. 10,791 pg/mL, *p* = 0.012). P-tau levels were markedly higher in MCI (A+) patients (106 pg/mL), whereas MCI (A−) patients had lower levels (37 pg/mL), followed by healthy controls (34 pg/mL). There was a significant difference between MCI (A+) and MCI (A−) patients (*p* < 0.001) and between MCI (A+) and healthy controls (*p* < 0.001).

Mean values of serum MDA had no significant difference between the study groups. Serum MDA levels were higher in MCI (A+) patients (209 ng/mL), followed by MCI (A−) patients (168 ng/mL) and healthy controls (166 ng/mL). Mean values of CSF MDA showed a significant difference between MCI (A+) and healthy controls (*p* = 0.024), and between MCI (A−) and healthy controls (*p* = 0.011). Healthy controls had markedly lower CSF MDA levels (83 ng/mL), whereas MCI (A+) had higher levels (130 ng/mL), followed by MCI (A−) patients (142 ng/mL).

Table 3 presents the mean values of CSF MDA and serum MDA in three groups, categorized by sex. In males, the mean values of serum MDA showed a significant difference between MCI (A+) patients and healthy controls (242 ng/mL vs. 146 ng/mL, *p* = 0.032). The mean values of CSF MDA showed a significant difference between MCI (A+) patients and healthy controls (175 ng/mL vs. 78 ng/mL, *p* = 0.012). In addition, CSF MDA levels were significantly different between MCI (A−) patients and healthy controls (142 ng/mL vs. 78 ng/mL, *p* = 0.048). In females, serum and CSF MDA levels did not differ significantly between the study groups. Serum MDA levels were higher in healthy controls (181 ng/mL), followed by MCI (A+) patients (180 ng/mL), and lower in MCI (A−) patients (135 ng/mL). CSF MDA levels were higher in MCI (A−) patients (142 ng/mL), followed by MCI (A+) patients (89 ng/mL), and lower in healthy controls (86 ng/mL).

Table 4 presents the mean values of CSF MDA and serum MDA in three groups, categorized by age. Individuals over 74 years old showed significantly higher serum MDA levels in MCI (A+) patients than in MCI (A−) patients (243 ng/mL vs. 152 ng/mL, *p* = 0.027). The mean values of CSF MDA did not reveal significant differences between the study groups. CSF MDA levels were higher in MCI (A−) patients (140 ng/mL), followed by MCI (A+) patients (128 ng/mL), and lower in healthy controls (83 ng/mL).

In individuals under 74 years old, the mean values of serum MDA did not reveal significant differences between the study groups. Seum MDA levels were in MCI (A−) patients (183 ng/mL), followed by healthy controls (168 ng/mL), and lower in MCI (A+) patients (141 ng/mL). CSF MDA showed a significant difference between MCI (A+) patients and healthy controls (135 ng/mL vs. 83 ng/mL, *p* = 0.047). In addition, CSF MDA levels were significantly different between MCI (A−) patients and healthy controls (145 ng/mL vs. 83 ng/mL, *p* = 0.011).

Table 5 shows the mean values of CSF MDA and serum MDA across three groups, based on education level. Individuals with a primary education level did not show significant differences in mean values of serum and CSF MDA between the study groups. Healthy controls had markedly lower serum MDA levels (86 ng/mL), whereas MCI (A+) had higher levels (165 ng/mL), followed by MCI (A−) patients (192 ng/mL). CSF MDA levels were higher in MCI (A−) patients (132 ng/mL), followed by healthy controls (111 ng/mL), and lower in MCI (A+) patients (88 ng/mL).

In terms of secondary education level, there were no significant differences in mean values of serum MDA between the study groups. Healthy controls had lower serum MDA levels (155 ng/mL), whereas MCI (A−) had higher levels (172 ng/mL), followed by MCI (A+) patients (242 ng/mL). CSF MDA levels were higher in MCI (A+) patients (173 ng/mL), followed by MCI (A−) patients (152 ng/mL), and markedly lower in healthy controls (86 ng/mL). The difference was significant between MCI (A+) and healthy controls (*p* = 0.045) in CSF MDA levels.

Individuals who had at least a tertiary education did not show significant differences in mean values of serum MDA between the study groups. Serum MDA levels were higher in MCI (A+) patients (198 ng/mL), followed by healthy controls (188 ng/mL), and lower in MCI (A−) patients (145 ng/mL). CSF MDA levels were higher in MCI (A−) patients (139 ng/mL), followed by MCI (A+) patients (112 ng/mL), and markedly lower in healthy controls (75 ng/mL). The difference was significant between MCI (A−) and healthy controls (*p* = 0.048) in CSF MDA levels.

## 4. Discussion

This study evaluated CSF biomarkers and MDA levels in adults aged 55–90, categorized into three groups: 30 MCI (A+) patients with abnormal levels of CSF Aβ (Group A), 30 MCI (A−) patients with normal levels of CSF Aβ (Group B), and 30 healthy (A−) participants with normal levels of CSF Aβ (Group C). Significant findings related to CSF biomarkers (Aβ42, Aβ42/Aβ40 ratio, and p-tau) and oxidative stress markers (MDA) provide further insights into their roles in cognitive decline and neurodegeneration.

The production and regulation of MDA in the central nervous system (CNS) are influenced by the unique environment of the CSF, which is more directly connected to neuronal activity and oxidative processes than serum. For instance, oxidative stress biomarkers, including MDA, better reflect the brain’s microenvironment when measured in CSF compared to peripheral blood. Since CSF is derived from plasma, it undergoes significant modification as it interacts with brain tissue, allowing it to better reflect local oxidative stress conditions [32]. In contrast, serum MDA levels may not show significant variation, as they are influenced by systemic factors that do not necessarily correlate with local CNS conditions [33].

This study found significant differences in CSF MDA levels between MCI (A+) and healthy individuals (A−), and between MCI (A−) and healthy individuals (A−), but not between MCI (A+) and MCI (A−) patients. This finding suggests that oxidative stress, as reflected by CSF MDA levels, may differentiate healthy aging from MCI, irrespective of amyloid status. However, no significant differences were observed in serum MDA levels across the groups, aligning with findings from recent studies [34,35] that show limited correlation between peripheral oxidative stress markers and neurodegenerative biomarkers in CSF. This discrepancy can be attributed to several factors related to the pathophysiology of MCI and the distinct roles of CSF and serum in reflecting oxidative stress and neuronal health.

In patients with MCI, particularly those with AD pathology, there is evidence suggesting that increased oxidative stress is associated with neurodegenerative processes. Elevated CSF MDA levels in these patients may indicate increased lipid peroxidation and neuronal damage, which are characteristic of neurodegenerative diseases [36]. In contrast, healthy individuals maintain a balance in oxidative stress biomarkers, as shown by the lower CSF MDA levels. The lack of significant differences in serum MDA levels across these groups suggests that systemic oxidative stress is not parallel with the localized oxidative processes occurring in the CNS of MCI patients [33].

Additionally, the role of astrocytes and other glial cells in modulating oxidative stress in the brain may also contribute to the observed differences in CSF MDA levels. Astrocytic dysfunction has been linked to altered CSF biomarker profiles, which may reflect the underlying neuroinflammatory processes associated with MCI. This dysfunction can lead to an imbalance in the production and clearance of reactive oxygen species (ROS), resulting in increased CSF MDA levels of MCI patients, while serum levels remain stable due to the buffering capacity of the peripheral system [37].

These results are consistent with evidence linking increased MDA levels to lipid peroxidation in MCI and AD [38,39]. Elevated MDA in plasma and CSF is a hallmark of oxidative stress in neurodegenerative diseases, while reduced antioxidant defenses, such as lower superoxide dismutase (SOD) levels, further exacerbate this process [26,40]. However, the absence of significant serum MDA differences in this study contrasts with prior reports linking higher serum MDA to cognitive impairment, including in post-stroke and atrial fibrillation patients [41,42].

Sex-stratified analysis revealed consistent differences in the MCI (A+) group. CSF MDA levels significantly differed between MCI (A+) and healthy individuals, as well as between males and females. Males with MCI exhibit significantly different CSF MDA levels compared to healthy individuals, while females do not show such differences. This agrees with previous findings, since males with MCI have been found to have significantly higher levels of MDA compared to age-matched controls, indicating a greater oxidative stress response that may contribute to their cognitive decline [40,43]. A recent study revealed that males may have a protective effect against MCI because of certain factors like lifestyle, genetic predispositions, or sociocultural factors, although there was no notable impact on dementia risk [16]. This could be the reason why increased lipid peroxidation occurs in the male population in the early phases of AD and in the progression of cognitive decline. In contrast, evidence regarding females with MCI and MDA levels remains inconclusive, with the study of Wang et al. indicating no significant differences [44].

Moreover, sex differences in the expression of certain biomarkers and genetic factors may also contribute to these discrepancies. For instance, the presence of the apolipoprotein E (APOE) ε4 allele has been associated with increased risk of AD and cognitive decline, and its effects may vary by sex. Research indicates that female APOE ε4 carriers show different patterns of CSF biomarkers compared to their male counterparts, particularly in the context of tau and amyloid levels [45]. This suggests that hormonal or genetic factors may modulate the oxidative stress response differently in males and females, leading to the observed differences in MDA levels.

Additionally, the role of neuroinflammation in cognitive impairment cannot be overlooked. Elevated levels of inflammatory markers have been associated with cognitive decline, and studies indicate that males may experience a more pronounced inflammatory response in the context of neurodegenerative diseases [46]. This increased inflammation could exacerbate oxidative stress, leading to increased MDA levels in males with MCI, while females may have a more resilient response to such stressors.

Age-stratified analysis revealed greater biomarker variability in individuals <74 years old compared to those ≥74 years old. Older participants demonstrated significant differences only in serum MDA levels between MCI (A+) and MCI (A−) patients, suggesting that oxidative stress biomarkers may play a more prominent role in younger individuals with MCI. This aligns with reports of increased oxidative stress in earlier stages of cognitive decline [47]. The statistically significant differences in CSF MDA levels between patients with MCI (A+) and healthy individuals (A−), and between MCI (A−) and healthy individuals (A−) in the age group of <74 years, but not in older individuals, can be attributed to several interrelated factors, including the dynamics of oxidative stress, the aging process, and the neurodegenerative changes associated with MCI.

Oxidative stress plays a critical role in the pathophysiology of cognitive decline. In younger individuals with MCI, elevated CSF MDA levels may reflect a more pronounced oxidative stress response, which could be linked to the early stages of neurodegeneration. The study of Liu et al. has shown that younger patients with MCI exhibit higher levels of oxidative stress markers, such as MDA, compared to healthy controls, indicating that the neurodegenerative processes may be more active or detectable at this age [43]. In contrast, older individuals may have a more stable oxidative stress profile due to the cumulative effects of aging, which could lead to a plateau in the levels of oxidative stress markers like MDA, thus masking differences between MCI patients and healthy controls [48].

Furthermore, aging is associated with various physiological changes that can influence oxidative stress levels. Antioxidant defenses may become less effective over time, leading to a different balance of oxidative stress and antioxidant capacity. In older adults, the body may adapt to chronic low-level oxidative stress, resulting in less variability in CSF MDA levels between those with MCI and healthy individuals [49]. This adaptation could explain why significant differences in CSF MDA levels are not observed in older age groups, as the oxidative stress response may not be as pronounced or may have reached a steady state.

The underlying mechanisms of neurodegeneration in MCI may also differ by age. Younger individuals with MCI may still exhibit significant neuroinflammatory responses and neuronal damage, which can be reflected in elevated CSF MDA levels. In contrast, older individuals may have more advanced neurodegenerative changes that are less sensitive to fluctuations in oxidative stress markers, as the neurodegenerative process may have already progressed to a stage where other biomarkers, such as tau or Aβ, become more relevant [50]. This shift in biomarker relevance could contribute to the lack of significant differences in CSF MDA levels among older adults with MCI.

In this study, MCI (A+) and MCI (A−) patients with secondary or tertiary education exhibited broader biomarker differences and higher levels of CSF MDA compared to primary education. This supports the hypothesis that cognitive reserve modulates the impact of neurodegenerative processes [38]. However, a recent study indicated education as a protective factor, with each additional year related to a 28% lower risk of MCI, whereas its effect on dementia remains unclear [16]. Other studies indicated that educational level significantly influences cognitive activity across the lifespan and is associated with a reduced risk of cognitive decline [17,18]. Further research is needed to investigate the effect of CSF MDA in MCI in terms of education level and other socioeconomic factors.

Some limitations apply to the present study. Firstly, it did not use the clinical classification based only on clinical assessments, into amnestic and non-amnestic MCI, which may have provided additional insights into biomarker variability across different MCI subtypes. Instead, we classified patients based on clinical assessments and laboratory FDA-approved CSF biomarkers. Secondly, the study lacked a follow-up period, preventing the assessment of longitudinal biomarker changes and their predictive value for cognitive decline. The relatively limited sample size may also restrict the generalizability of the findings, particularly in subgroup analyses based on sex, age, and education level. Future research should address these limitations by incorporating a broader spectrum of MCI cases, longitudinal assessments, and larger, more diverse cohorts to further elucidate the complex interplay between oxidative stress and neurodegeneration.

The findings highlight the relevance of CSF MDA as a marker of oxidative stress in neurodegeneration. However, the absence of significant serum MDA differences limits its utility as a peripheral biomarker, echoing previous reports [35,51]. The interplay between oxidative stress, amyloid, and tau pathology, influenced by demographic factors such as sex, age, and education level, underscores the complexity of neurodegenerative diseases. Future research should explore longitudinal changes in these biomarkers, incorporate antioxidant defenses, and expand the analysis to larger, diverse cohorts to enhance our understanding of these interrelationships.

## 5. Conclusions

This study highlights the importance of CSF MDA as a potential marker of oxidative stress, while serum MDA showed limited utility. CSF MDA levels are higher in MCI (A−) patients within the study groups, suggesting their potential use as an early biomarker to distinguish MCI patients. Demographic factors such as sex, age, and education level significantly influenced biomarker expression, emphasizing the need for personalized approaches in biomarker analysis and interpretation. These results contribute to the growing body of evidence supporting the use of FDA-approved biomarkers and oxidative stress markers in understanding and differentiating neurodegenerative processes. Future studies should aim to validate these findings in larger cohorts and explore their implications for therapeutic interventions and disease monitoring.

## Figures and Tables

**Table 1:** Characteristics of the study groups.

		Characteristics of the Study Groups		
		Group A (n = 30) MCI (A+)	Group B (n = 30) MCI (A−)	Group C (n = 30) Controls
Sex	Male (n)	14.0	16.0	12.0
	Female (n)	16.0	14.0	18.0
Age	≥74 years old (n)	16.0	15.0	12.0
	<74 years old (n)	14.0	15.0	18.0
BMI	kg/m^2^	26.5	27.4	28.2
Education level	Primary (n)	6.0	8.0	4.0
	Secondary (n)	12.0	11.0	8.0
	Tertiary (n)	12.0	11.0	18.0
Family history	Yes (n)	14.0	21.0	13.0
	No (n)	16.0	9.0	17.0

Abbreviations: BMI: body mass index.

**Table 2:** Mean values with the corresponding results of the *t*-test for mean comparisons of the CSF FDA-approved biomarkers and MDA within the study groups.

	Mean Values ± SD			*p*-Values ^a^		
Cerebrospinal Fluid Biomarkers	Group A MCI (A+) (n = 30)	Group B MCI (A−) (n = 30)	Group C Controls (n = 30)	Group A vs. Group B	Group A vs. Group C	Group B vs. Group C
Aβ42 (pg/mL)	590 ± 200	1004 ± 461	1237 ± 332	<0.001	<0.001	0.015
Aβ40 (pg/mL)	13,087 ± 4293	10,791 ± 4066	12,017 ± 2480	0.012	0.124	0.085
Aβ42/Aβ40 ratio	0.05 ± 0.007	0.09 ± 0.02	0.10 ± 0.015	<0.001	<0.001	0.008
p-tau (pg/mL)	106 ± 51	37 ± 16	34 ± 8.78	<0.001	<0.001	0.157
t-tau (pg/mL)	633 ± 234	314 ± 95	252 ± 66	<0.001	<0.001	0.007
MDA						
Serum (ng/mL)	209 ± 120	168 ± 191	166 ± 159	0.159	0.124	0.488
CSF (ng/mL)	130 ± 106	142 ± 118	83 ± 70	0.345	0.024	0.011

Abbreviations: SD: standard deviation, Aβ: amyloid beta, p-tau: phosphorylated tau protein, t-tau: total tau protein, MDA: malondialdehyde, CSF: cerebrospinal fluid. Note: ^a^ *t*-test for independent samples.

**Table 3:** Mean values with the corresponding results of the *t*-test for mean comparisons of MDA within the study groups, according to sex.

	**Males** **(Mean Values ± SD)**			***p*-Values ^a^**		
**MDA**	**Group A** **MCI (A+)** **(n = 14)**	**Group B** **MCI (A−)** **(n = 16)**	**Group C** **Controls** **(n = 12)**	**Group A** **vs.** **Group B**	**Group A** **vs.** **Group C**	**Group B** **vs.** **Group C**
Serum (ng/mL)	242 ± 120	196 ± 205	146 ± 165	0.232	0.032	0.247
CSF (ng/mL)	175 ± 125	142.5 ± 116	78 ± 77	0.236	0.012	0.048
	**Females** **(Mean Values ± SD)**			***p*-Values ^a^**		
	**Group A** **MCI (A+)** **(n = 16)**	**Group B** **MCI (A−)** **(n = 14)**	**Group C** **Controls** **(n = 18)**			
Serum (ng/mL)	180 ± 116	135 ± 175	181 ± 159	0.206	0.493	0.228
CSF (ng/mL)	89 ± 64	142 ± 126	86 ± 67	0.082	0.454	0.065

Abbreviations: SD: standard deviation, MDA: malondialdehyde, CSF: cerebrospinal fluid. Note: ^a^ *t*-test for independent samples.

**Table 4:** Mean values with the corresponding results of the *t*-test for mean comparisons of MDA within the study groups, according to age.

	**Aged ≥ 74** **(Mean Values ± SD)**			***p*-Values ^a^**		
**MDA**	**Group A** **MCI (A+)** **(n = 16)**	**Group B** **MCI (A−)** **(n = 15)**	**Group C** **Controls** **(n = 12)**	**Group A vs.** **Group B**	**Group A** **vs.** **Group C**	**Group B** **vs.** **Group C**
Serum (ng/mL)	243 ± 100	152 ± 189	157 ± 140	0.027	0.078	0.481
CSF (ng/mL)	128 ± 115	140 ± 141	83 ± 63	0.397	0.232	0.227
	**Aged < 74** **(Mean Values ± SD)**			***p*-Values ^a^**		
	**Group A** **MCI (A+)** **(n = 14)**	**Group B** **MCI (A−)** **(n = 15)**	**Group C** **Controls** **(n = 18)**			
Serum (ng/mL)	141 ± 131	183 ± 183	168 ± 165	0.279	0.325	0.395
CSF (ng/mL)	135 ± 91	145 ± 96	83 ± 72	0.403	0.047	0.011

Abbreviations: SD: standard deviation, MDA: malondialdehyde, CSF: cerebrospinal fluid. Note: ^a^ *t*-test for independent samples.

**Table 5:** Mean values with the corresponding results of the *t*-test for mean comparisons of MDA within the study groups, according to education level.

**Primary Education Level**						
	**Mean Values ± SD**			***p*-Values ^a^**		
**MDA**	**Group A** **MCI (A+)** **(n = 6)**	**Group B** **MCI (A−)** **(n = 8)**	**Group C** **Controls** **(n = 4)**	**Group A** **vs.** **Group B**	**Group A** **vs.** **Group C**	**Group B** **vs.** **Group C**
Serum (ng/mL)	165 ± 123	192 ± 207	86 ± 103	0.393	0.158	0.181
CSF (ng/mL)	88 ± 42	132 ± 128	111 ± 91	0.219	0.298	0.389
**Secondary Education Level**						
	**Mean Values ± SD**			***p*-Values ^a^**		
	**Group A** **MCI (A+)** **(n = 12)**	**Group B** **MCI (A−)** **(n = 11)**	**Group C** **Controls** **(n = 8)**			
Serum (ng/mL)	242 ± 132	172 ± 259	155 ± 132	0.209	0.093	0.438
CSF (ng/mL)	173 ± 84	152 ± 124	86 ± 62	0.342	0.045	0.132
**Tertiary Education Level**						
	**Mean Values ± SD**			***p*-Values ^a^**		
	**Group A** **MCI (A+)** **(n = 12)**	**Group B** **MCI (A−)** **(n = 11)**	**Group C** **Controls** **(n = 18)**			
Serum (ng/mL)	198 ± 105	145 ± 87	188 ± 178	0.106	0.436	0.230
CSF (ng/mL)	112 ± 135	139 ± 93	75 ± 71	0.295	0.166	0.048

Abbreviations: SD: standard deviation, MDA: malondialdehyde, CSF: cerebrospinal fluid. Note: ^a^ *t*-test for independent samples.

## Data Availability

The original contributions presented in this study are included in the article. Further inquiries can be directed to the corresponding author.

## Abbreviations

Aβ	Amyloid β
AD	Alzheimer’s disease
BMI	Body mass index
CSF	Cerebrospinal fluid
CNS	Central nervous system
FDA	Food and Drug Administration
GAADRD	Greek Association of Alzheimer’s Disease and Related Disorders
MCI	Mild Cognitive Impairment
MDA	Malondialdehyde
p-tau	Phosphorylated tau protein
t-tau	Total tau protein
SD	Standard deviation
